# The legacy of Bruce Ames and mitochondrial DNA mutagenicity: integrating oxidative stress, aging, and modern perspectives

**DOI:** 10.3389/fmolb.2025.1710944

**Published:** 2025-10-29

**Authors:** Jennifer Dang, Jia Yu Liou, Rida Mullah, Cecilia Giulivi

**Affiliations:** ^1^ Department of Molecular Biosciences, School of Veterinary Medicine, University of California Davis, Davis, CA, United States; ^2^ MIND Institute, University of California at Davis Medical Center, Sacramento, CA, United States

**Keywords:** mtDNA mutagenesis, oxidative stress, Ames bacterial mutagenicity test, triage theory, mitochondrial dysfunction, aging, nutrition

## Introduction

**FIGURE 1:**
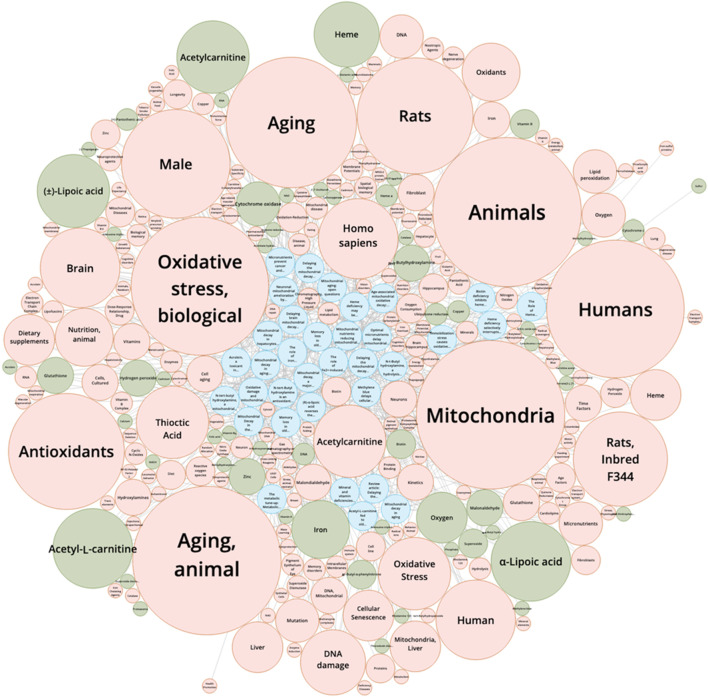
Knowledge Graph Mapping from References. This knowledge graph visualizes the interconnections between key references (articles only), chemical substances, and scientific concepts derived from a SciFinder literature search using the keywords *Bruce Ames*, *mitochondria*, and *aging*. Blue nodes represent primary references, highlighting seminal papers such as Ames (1973, 1993) and subsequent studies on mitochondrial mutagenicity and aging. Green nodes correspond to chemical substances, including antioxidants and micronutrients relevant to Ames’s triage theory and mitochondrial oxidative stress. Orange nodes indicate scientific concepts, such as oxidative stress and aging mechanisms. The edges connecting nodes depict relationships extracted from co-occurrence patterns in the literature, illustrating how Ames’s work intersects with chemical modulators and mechanistic pathways in mitochondrial biology. This figure provides an integrated overview of the interrelationships between studies on Bruce Ames, mitochondrial function, and aging, revealing clusters of high-impact references, commonly studied compounds, and central conceptual themes in the field. Bibliographic and chemical data used to generate this knowledge graph were obtained from SciFinder (CAS, American Chemical Society). The figure was redistributed with permission. Copyright ^©^ 2025 the American Chemical Society (ACS). All rights reserved.

Bruce Ames stands among the most influential biochemists of the past half-century, leaving a profound mark on genetics, toxicology, nutrition, and aging research. Best known for developing the *Ames test*, a bacterial assay that detects mutagenic chemicals, Ames helped establish the principle that environmental mutagens can be reliably measured and systematically screened (Ames, 1973). This transformed toxicology and cancer prevention, saving countless lives by enabling the regulation of carcinogens. Yet Ames’s legacy extends beyond mutagen detection. In the latter part of his career, he focused on oxidative stress and mitochondrial DNA (mtDNA) mutagenicity as central players in the biology of aging (Ames et al., 1993). While the foundational ideas of oxidative damage and mitochondrial contribution to aging were first proposed by researchers such as Gershman and Harman, Ames contributed by integrating these concepts with experimental observations on mitochondrial decay, nutrition, and disease. He highlighted how reactive oxygen species (ROS) produced during mitochondrial respiration could contribute to cumulative mtDNA damage, potentially impairing mitochondrial function over time. Ames also emphasized the role of micronutrients in modulating this process, proposing that suboptimal nutrition could accelerate oxidative damage and mitochondrial decline through his triage theory (Ames, 2006) (Figure 1). His work helped translate earlier theoretical ideas into a broader framework linking oxidative stress, mitochondrial function, and age-related health outcomes.

While this concept was groundbreaking, the field has since evolved. Contemporary research has revealed a more complex picture, with some scientists challenging the causal role of mtDNA damage in aging, proposing instead that mitochondrial dysfunction may arise from signaling changes, metabolic imbalances, or programmed processes rather than cumulative mutations (Bratic and Larsson, 2013). This paper will examine Bruce Ames’s contributions, with particular focus on mtDNA mutagenicity, and critically assess how his theories shaped — and continue to spark debate within — modern biogerontology.

## The Ames test and the assessment of mutagenicity

In the 1970s, Ames introduced a simple yet powerful tool for detecting chemical mutagens: a bacterial assay using strains of *Salmonella typhimurium* engineered to be highly sensitive to DNA mutations. If exposure to a chemical restored growth via human or rat liver-activated metabolism in these bacteria (via reversion mutations), the compound was considered mutagenic. Importantly, Ames demonstrated that many environmental chemicals, including industrial pollutants, food additives, and pesticides, scored positive in the assay — and many of these same compounds later proved carcinogenic in animals (Ames et al., 1973).

The test revolutionized toxicology by offering a rapid, inexpensive alternative to long-term rodent studies. Regulatory agencies worldwide adopted it to screen thousands of compounds, dramatically reducing public exposure to carcinogens. The “Ames test” also established a broader principle: genetic mutagenesis underpins carcinogenesis, and therefore, mutagens can be used as predictors of cancer risk. This work reflected Ames’s larger intellectual project: to bridge laboratory assays with real-world human health, from chemical safety to disease prevention.

## A shift toward oxidative stress and mtDNA damage

By the 1980s, Ames expanded his focus from exogenous mutagens (environmental chemicals) to endogenous sources of DNA damage within the cell. He became especially interested in mitochondria, the cellular organelles that generate energy but also produce ROS as a byproduct of oxidative phosphorylation (Aliev et al., 2009; Ames, 1983; Ames, 1989; Ames, 2005; Ames, 2010; Ames, 2018; Ames et al., 1991; Ames et al., 1995; Atamna et al., 2002a; Atamna et al., 2001a; Atamna et al., 2007; Atamna et al., 2008; Atamna et al., 2000; Atamna et al., 2001b; Atamna et al., 2002b; Beckman and Ames, 1999; Chen et al., 1995; Gogvadze et al., 2003; Hagen et al., 1999; Hagen et al., 1998; Hagen et al., 2002; Hagen et al., 2000; Hagen et al., 1997; Helbock et al., 1998; Jia et al., 2007; Killilea and Ames, 2008; Killilea et al., 2003; Lal et al., 2008; Liu et al., 2002a; Liu et al., 2002b; Liu et al., 1996; Liu et al., 2000; Long et al., 2009; Lykkesfeldt et al., 1998; Milgram et al., 2007; Shenk et al., 2009; Voloboueva et al., 2007; Voloboueva et al., 2005; Walter et al., 2002; Walter et al., 2013; Yowe and Ames, 1998).

Unlike nuclear DNA, mtDNA is highly vulnerable. It is located near the electron transport chain, where ROS are generated, lacks protective histones, and has more limited repair mechanisms. Ames proposed that cumulative mtDNA mutations lead to a decline in mitochondrial efficiency, greater ROS leakage, and a vicious cycle of escalating damage (Ames et al., 1993). This “mitochondrial decay” hypothesis became central to his later research, with implications for cancer, neurodegeneration, cardiovascular disease, and aging.

Ames further argued that nutrition plays a key role in modulating this process (Aliev et al., 2009; Ames, 1983; Ames, 2010; Ames, 2018; Hagen et al., 1999; Hagen et al., 1998; Hagen et al., 2002; Hagen et al., 2000; Liu et al., 2002b; Long et al., 2009; Milgram et al., 2007; Shenk et al., 2009; Voloboueva et al., 2005). His “triage theory” suggested that when micronutrients (such as vitamins, minerals, and antioxidants) are limited, the body prioritizes short-term survival functions over long-term DNA maintenance. As a result, suboptimal nutrition accelerates oxidative damage, hastening mitochondrial decline and aging (Ames, 2006). An example of how modest micronutrient deficiencies can lead to long-term damage is illustrated in Ames’ work on iron deficiency. Iron, an essential trace mineral for iron-sulfur clusters and heme synthesis, enables complex function in the electron transport chain. Ames posits that when iron is limited, decreased heme and iron-sulfur cluster availability impairs electron transport and causes mitochondrial uncoupling and superoxide release (Walter et al., 2002). If iron deficiency is persistent over time, the resulting ROS leads to ongoing oxidative stress, mtDNA damage, and mitochondrial decline associated with age. Subsequently, if iron deficiency is exacerbated, the preceding symptoms lead to iron deficiency anemia (Pau et al., 2017). This framework, applied to other micronutrients, has elevated nutrition and metabolism as key determinants of genomic stability and longevity.

The work of Dr. Ames can be cross-referenced with that of our team, which has also extensively studied mitochondrial dysfunction in the context of aging and neurodegeneration. However, our focus is more disease-specific. While Dr. Ames emphasized systemic mitochondrial decay and the role of oxidative damage in aging and age-related diseases (Ames et al., 1993), our research explores mitochondrial bioenergetics, redox balance, and apoptotic pathways in long-lived or post-mitotic cells, particularly neurons, in conditions such as Huntington’s disease, fragile X-associated tremor/ataxia syndrome, and schizophrenia (Giulivi et al., 2010; Napoli et al., 2016; Song et al., 2016; Napoli et al., 2013). Together, our works highlight the centrality of mitochondria in both age-related and disease-specific cellular decline. Ames’s systemic and nutritional perspective complements our team’s mechanistic and cell-specific insights, suggesting that interventions aimed at preserving mitochondrial function could benefit both healthy aging and the mitigation of neurodegenerative disorders.

## Early theoretical and experimental links between mtDNA damage and aging

The idea that mitochondrial DNA mutations contribute to aging did not originate solely with Bruce Ames. Earlier work laid critical foundations. In the 1950s, Rebecca Gershman was the first to suggest that oxygen- and nitrogen-centered free radicals could play a role in biological damage, a radical departure from the prevailing view that oxygen was purely beneficial (Gerschman et al., 1954). Building on this, Denham Harman proposed the *Free Radical Theory of Aging* in 1956, later refining it in 1972 into the *Mitochondrial Free Radical Theory of Aging (MFRTA)*. Harman argued that mitochondria are both generators and victims of reactive oxygen species, and that cumulative oxidative damage leads to cellular dysfunction and senescence (Harman, 1972). Ames and his colleagues played a significant role in developing and advancing the theory. In the mid-90s, Ames published research showing that mitochondrial function declines with age and that this decay could be mitigated in rats with certain micronutrients or antioxidants such as acetyl-L-carnitine, α-lipoic acid, and *N*-tert-butylhydroxylamine (Atamna et al., 2001b; Hagen et al., 2002; Shigenaga et al., 1994). While not the originator of the idea, Ames’s work provided crucial experimental evidence and added significant detail to the understanding of mitochondrial decline in aging.

Further experimental support for these ideas began to emerge in the 1980s. Pikó and colleagues (1988) provided some of the earliest evidence by reporting increased deletions in the mtDNA of aged rodents, linking mitochondrial genomic instability to aging phenotypes (Piko et al., 1988). This strengthened the hypothesis that mtDNA integrity was a critical factor in age-related decline.

More decisive evidence arrived in the early 2000s with the development of *mtDNA mutator mice* (Trifunovic et al., 2004). These mice carried mutations in the mitochondrial DNA polymerase gamma (*Polg*) that caused an accelerated accumulation of mtDNA mutations. The animals exhibited a progeroid syndrome — premature aging features such as hair loss, osteoporosis, and reduced lifespan. This was widely interpreted as proof that increased mtDNA mutation burden could drive aging, offering direct experimental validation of earlier theoretical models (Trifunovic et al., 2004).

Ames’s later work on oxidative stress and mitochondrial decay fit squarely within this trajectory. While Gershman and Harman framed the initial conceptual theories and studies, like Pikó’s and the mutator mice, provided experimental support, Ames helped integrate these ideas into a broader picture of mutagenesis, nutrition, and public health, making the subject accessible across disciplines.

## Mitochondrial mutagenesis and the biology of aging

The idea that mtDNA damage drives aging resonated deeply within biogerontology. For decades, the “oxidative stress theory of aging” was one of the most widely accepted models of senescence. Numerous studies have appeared to support this: aged tissues exhibit higher levels of mtDNA mutations, dysfunctional mitochondria, and oxidative stress biomarkers. In animal models, interventions that boosted antioxidant defenses or enhanced mitochondrial function sometimes extended lifespan or delayed age-related decline (Shigenaga et al., 1994).

Ames’s work thus provided both a mechanistic hypothesis and a therapeutic rationale: reduce oxidative damage, protect mtDNA, and thereby slow aging. This vision helped catalyze entire subfields focused on antioxidants, caloric restriction mimetics, and mitochondrial-targeted therapies.

## Challenges and current understanding of mitochondria in aging

Despite the compelling evidence that oxidative stress and mtDNA mutations contribute to cellular decline, more recent research has revealed that the relationship between mitochondria and aging is far more complex than initially proposed. For example, interventions with antioxidants, while effective at reducing oxidative markers, have generally failed to extend lifespan in both human or animal studies due to antioxidants disrupting crucial oxidant signaling (Ristow and Schmeisser, 2011). This suggests that reactive oxygen species (ROS) are not simply damaging agents; at low levels, they may play important signaling roles that promote cellular adaptation and survival, a concept now referred to as *mitohormesis* (Ristow and Schmeisser, 2011).

Although mtDNA mutations do accumulate with age, the actual mutation burden in many tissues remains relatively low (Larsson, 2010) and often insufficient to explain widespread age-related physiological decline. Some aged individuals maintain robust mitochondrial function despite the presence of detectable mtDNA mutations, suggesting that mitochondrial DNA damage alone is not the sole driver of aging (Larsson, 2010). Mouse models with artificially accelerated mtDNA mutations, such as *Polg* mutator mice, display progeroid phenotypes (Trifunovic et al., 2004). Still, the levels of mutation in these models far exceed those found in natural aging, indicating that these experimental systems may exaggerate the impact of mtDNA mutations (Trifunovic et al., 2004).

Current research has shifted toward a more nuanced view of mitochondrial aging, emphasizing that dysfunction arises from multiple interconnected mechanisms. Epigenetic regulation, altered signaling (e.g., NAD^+^/sirtuins), proteostasis, and changes in mitochondrial dynamics all contribute to aging more broadly than just impaired ATP production (Sun et al., 2016; Budinger and Chandel, 2025). With age, the balance between mitochondrial fission and fusion is disrupted, and defective mitochondria that should be cleared by mitophagy accumulate, driving cellular dysfunction. While mitochondrial function may decline in sedentary individuals, evidence suggests this decline is not inevitable, as maintaining an active lifestyle can preserve mitochondrial efficiency and energy metabolism.

Importantly, the effects of aging on mitochondrial function are highly cell-type and tissue-specific. A 2024 study (Ehinger et al., 2024) found only minor, largely insignificant changes in mitochondrial respiration in blood cells across the human lifespan. In contrast, other research examining long-lived, differentiated, or non-dividing cells—including neurons, cardiomyocytes, and skeletal muscle fibers—has documented substantial declines in mitochondrial function, including reduced ATP production, altered membrane potential, and impaired oxidative capacity (Chubanava et al., 2025; Peterson et al., 2012; Boengler et al., 2017; Johnson et al., 2013; Seo et al., 2016; Tocchi et al., 2015; Marzetti et al., 2013; Short et al., 2005; Sagar and Gustafsson, 2023; Li et al., 2023; Jeong et al., 2024; Chen et al., 2023; Grevendonk et al., 2021; Harper et al., 2021; Kamarulzaman and Makpol, 2025; Nuccio et al., 2024; Tepp et al., 2016; Wang et al., 2025; Zhang et al., 2025; Springer-Sapp et al., 2025; Hepple, 2014). These observations indicate that mitochondrial aging is not uniform throughout the body, and specific post-mitotic cells may be particularly susceptible to dysfunction.

Lifestyle factors, particularly physical activity, also play an essential role in modulating mitochondrial aging. Evidence suggests that many of the declines observed in sedentary individuals reflect reduced activity rather than inevitable aging itself. This distinction is underscored by findings in animal models, where dietary restrictions and moderate physical exercise enhanced mitochondrial activity and decreased ROS formation (Ristow and Schmeisser, 2011).

Taken together, these findings challenge the traditional view that oxidative damage and mtDNA mutations alone are the primary drivers of aging. Instead, mitochondrial dysfunction appears to be multifactorial, involving disrupted signaling, imbalanced dynamics, impaired quality control, lifestyle influences, and tissue- and cell-type specific vulnerabilities. This contemporary understanding complements and refines Ames’s original hypotheses, highlighting the complexity of mitochondrial biology and emphasizing that interventions targeting signaling pathways, network dynamics, and lifestyle factors may be more effective than strategies that focus exclusively on preventing oxidative damage or mutagenesis.

## The enduring influence of Professor Bruce Ames

Even with these challenges, Ames’s influence remains profound. His work continues to shape scientific and public discourse in several ways:

• Methodological impact: The Ames test remains a staple in toxicology, underscoring its lasting contribution to public health.
• Conceptual legacy: By linking mutagenesis, mitochondrial biology, and nutrition, Ames helped frame aging as a mechanistic, molecular process rather than an inevitable, mysterious decline.
• Nutritional insights: His triage theory broadened how scientists think about micronutrient deficiencies, suggesting that even marginal deficits can accelerate long-term genomic instability (Ames, 2006).
• Catalyst for new theories: Even as the oxidative stress model is revised, the debates it inspired have fueled breakthroughs in mitohormesis, mitochondrial signaling, and metabolic regulation.

In this sense, the critiques of Ames’s model are not rejections but evolutions — refining the questions he first posed about damage, repair, and aging.

## Conclusion

Bruce Ames’s career illustrates how a scientist’s influence can transcend specific findings. His Ames test forever changed the field of chemical safety and mutagen screening. At the same time, his later work on oxidative stress and mtDNA mutagenicity provided a powerful, albeit imperfect, framework for understanding aging. Although modern evidence challenges the view that accumulated mtDNA mutations are the central drivers of aging, Ames’s theories catalyzed decades of research that continue to shape biogerontology. Today, as scientists explore mitochondrial signaling, metabolic pathways, and the complex interplay between genetics and environment, they build on the foundation Ames helped create. His legacy is therefore not tied to a single test or theory, but to a larger vision: that careful mechanistic science, applied to the most fundamental processes of biology, can illuminate the path to better health and longer life.
